# Supplementary material for: Identification of CD133+ intercellsomes in intercellular communication to offset intracellular signal deficit
Source: eLife. 2023 Oct 17;12:RP86824. doi: 10.7554/eLife.86824 (PMC10581692; doi:10.7554/eLife.86824)
Supplement: Figure 3—figure supplement 2—source data 1. [file elife-86824-fig3-figsupp2-data1.pdf]

Figure 3-figure supplement 2; western blot

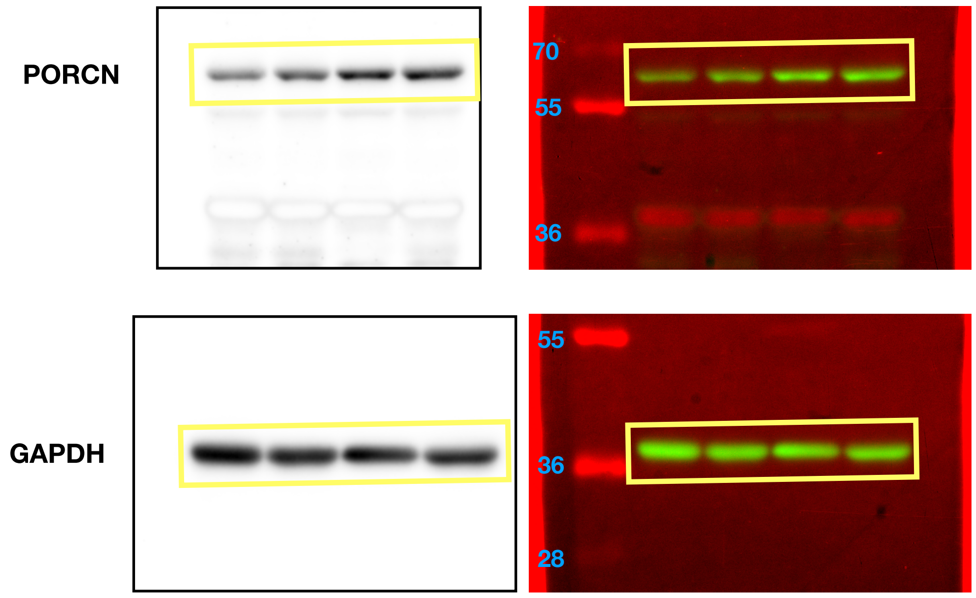

Only one linear adjustment was performed from raw data to figures (including inside the imaging instrument)
