# Supplementary material for: Identification of CD133+ intercellsomes in intercellular communication to offset intracellular signal deficit
Source: eLife. 2023 Oct 17;12:RP86824. doi: 10.7554/eLife.86824 (PMC10581692; doi:10.7554/eLife.86824)
Supplement: Figure 4—source data 1. [file elife-86824-fig4-data1.pdf]

Fig. 4; western blot

Panel E, PLC

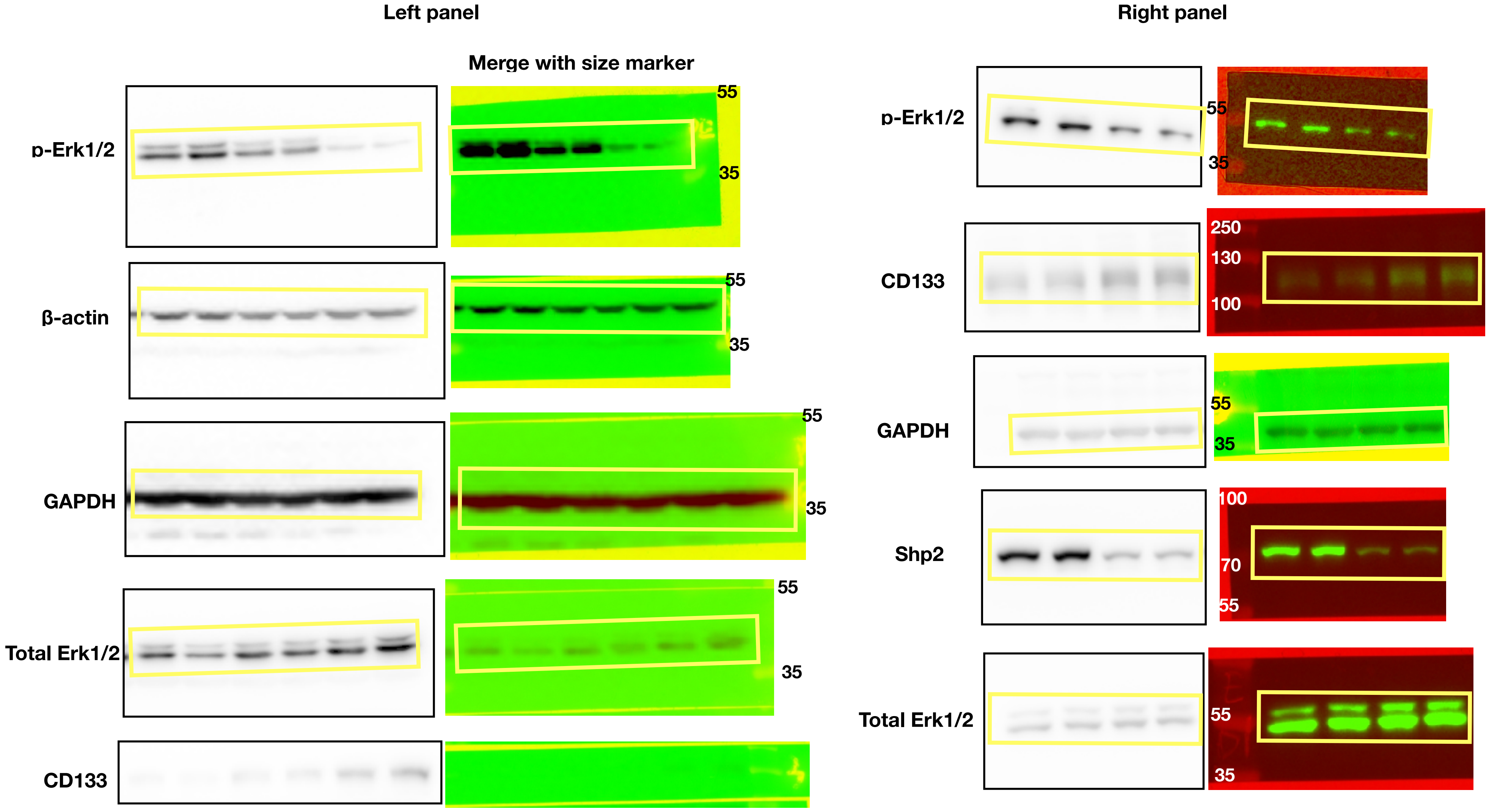

Panel G, MCF10A

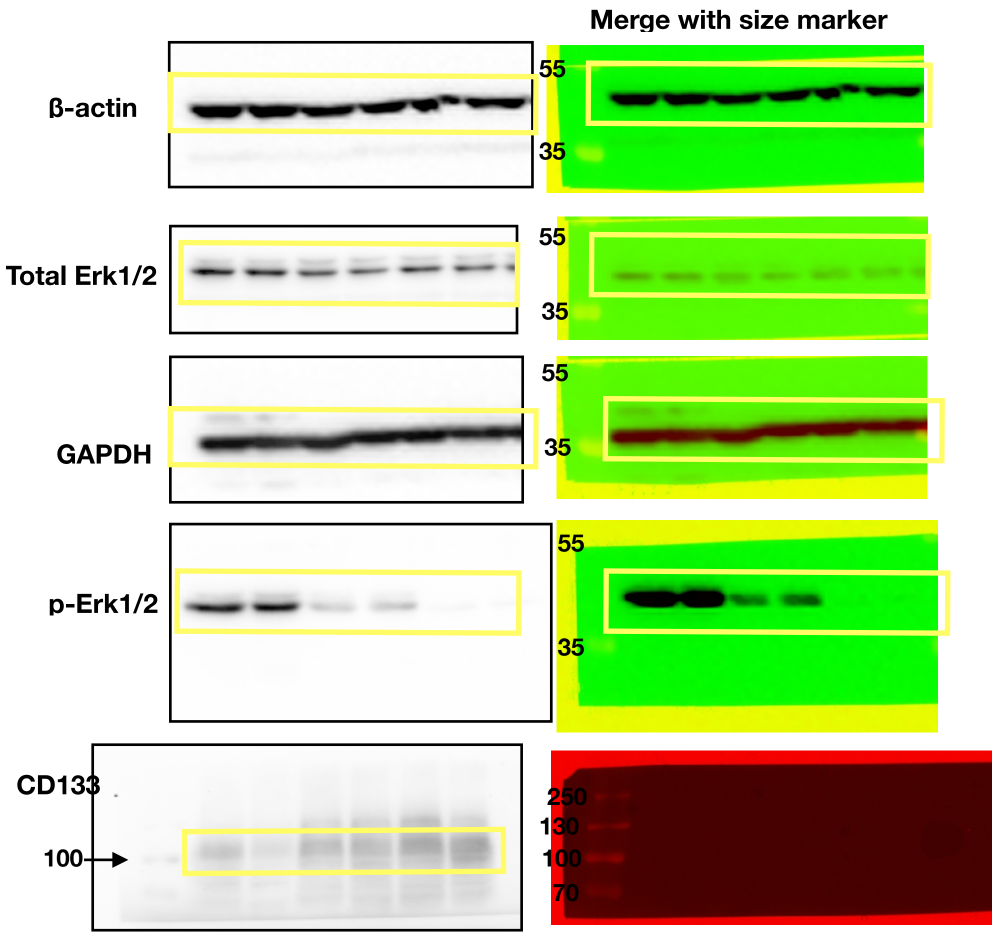

Only one linear adjustment was performed from raw data to figures (including inside the imaging instrument)
