# Supplementary material for: Identification of CD133+ intercellsomes in intercellular communication to offset intracellular signal deficit
Source: eLife. 2023 Oct 17;12:RP86824. doi: 10.7554/eLife.86824 (PMC10581692; doi:10.7554/eLife.86824)
Supplement: Figure 7—source code 1. [file elife-86824-fig7-code1.zip › README.html]

RNA exchange simulation


# RNA exchange simulation

```

## Description

This function is used to perform RNA exchange simulation among CD133- hepatocytes. The basic idea was to randomly divide all CD133- hepatocyes into small groups with size N and simulate that cells within each group exchange IEGs with each other. The simulation results are then visualized and evaluated by plotting IEG diversity (entropy) against total IEG expression levels.

## Expected output

A data.frame with meta cell IEG diversity (entropy) and total IEG expression levels.

## Example

```
# Import data and function
source('Simulation.R')

normalized.dat <- readRDS('Normalized.dat.Rds')
IEG_mean <- readRDS('IEG_mean_expression.Rds')
Entropy <- readRDS('Entropy.Rds')
Meta_cell <- readRDS('Meta_cell.Rds')
Seeds <- readRDS('Seeds.Rds')

IEG <- read.delim('Liver_IEG.txt')
as.character(IEG$GeneName)
```

```
##  [1] "Fos"  "Jun"  "Egr1" "Ier2" "Atf3" "Junb" "Myc"  "Crem" "Ets2" "Ier3"
## [11] "Lepr" "Egfr"
```

```
# Run function
res_sim <- exchange_simulation(normalized.data = normalized.dat, seeds = Seeds,
                               Entropy = Entropy, IEG_mean_total = IEG_mean,
                               size = 5, ex = 1/12, ex2 = 1/12, mode = 3, n_meta = 50, 
                               col_name = "Neg_sim")
```

```
# Visualization
library(ggplot2)
colnames(res_sim) <- c('Entropy_sim', 'CyclinD1', 'CD133', 'IEG_sim')
res_Pos <- Meta_cell[Meta_cell$CD133 == 'Pos', ]
res_Neg <- Meta_cell[Meta_cell$CD133 == 'Neg', ]
res_Neg_ori_vs_sim <- cbind(res_Neg, res_sim[,c(1,4)])
ggplot() + 
        geom_point(data = res_Neg_ori_vs_sim, aes(x=IEG, y=Entropy), color='black') +
        geom_point(data = res_Neg_ori_vs_sim, aes(x=IEG_sim, y=Entropy_sim), color='red') +
        geom_point(data=res_Pos, aes(x=IEG, y=Entropy), color='olivedrab2', linetype = "dashed", size=4)+
        geom_path(data=res_Pos, aes(x=IEG, y=Entropy), color='olivedrab2', linetype = "dashed", size=1) +
        geom_segment(data = res_Neg_ori_vs_sim, aes(x=IEG, y=Entropy, xend=IEG_sim, yend=Entropy_sim),
                     arrow=arrow(length = unit(0.15, "cm")),color='gray60',size=0.5,linejoin='round') +
        xlab('IEG') +
        ylab('Entropy') +
        theme(axis.text=element_text(size=12, face="bold"), axis.title=element_text(size=14,face="bold"))
```

## Reference

Use zero-inflated Negative Binomial (ZINB) regression for modeling single-cell RNAseq data:

Miao, Z., Deng, K., Wang, X. & Zhang, X. DEsingle for detecting three types of differential expression in single-cell RNA-seq data. Bioinformatics 34, 3223-3224, doi:10.1093/bioinformatics/bty332 (2018).
