# Supplementary material for: Identification of CD133+ intercellsomes in intercellular communication to offset intracellular signal deficit
Source: eLife. 2023 Oct 17;12:RP86824. doi: 10.7554/eLife.86824 (PMC10581692; doi:10.7554/eLife.86824)
Supplement: Figure 7—source data 1. [file elife-86824-fig7-data1.pdf]

Fig. 7; western blot

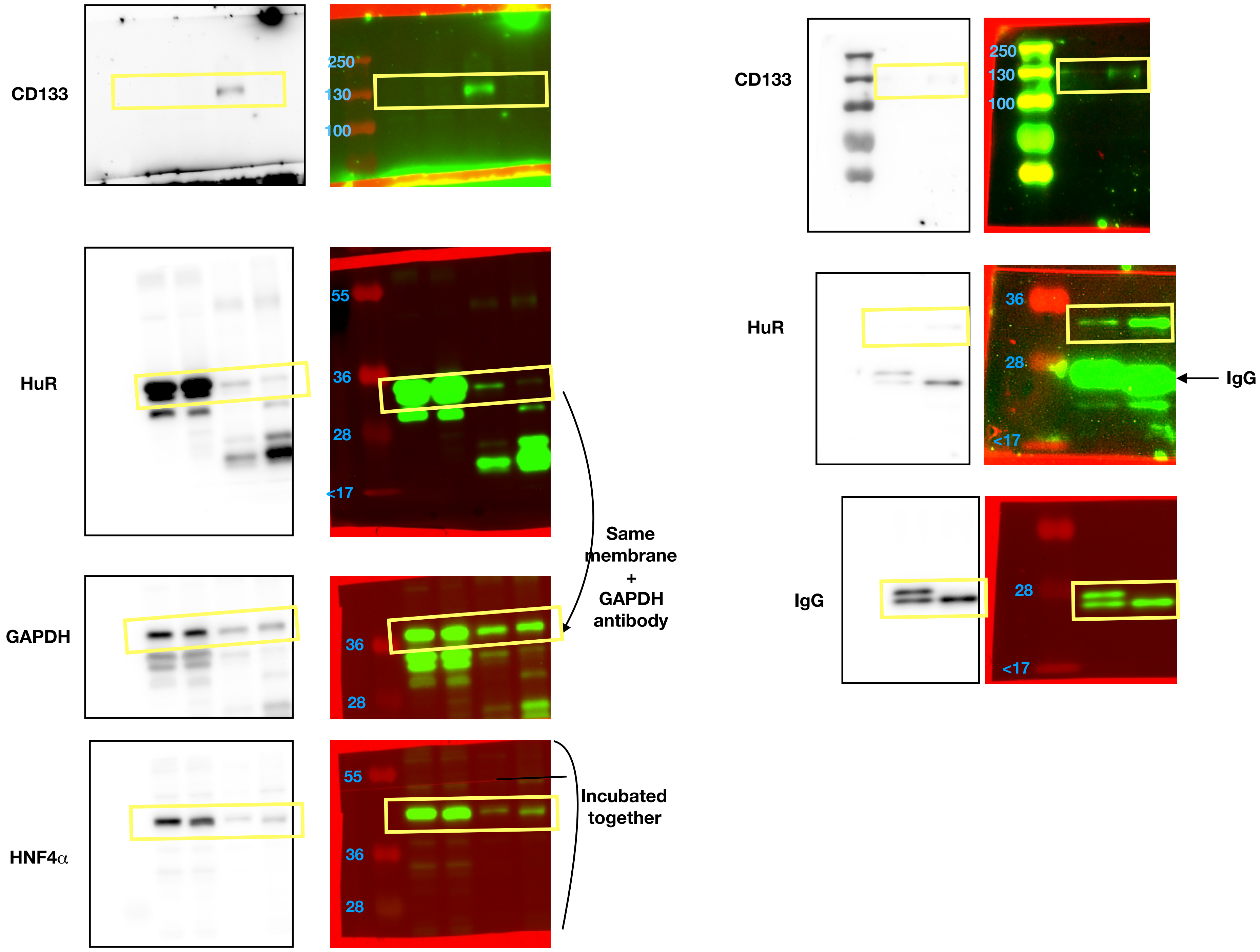

Only one linear adjustment was performed from raw data to figures (including inside the imaging instrument)

Due to the limited amount of the protein lysates (isolated vesicles), membranes were cut into multiple pieces and incubated for different antibodies. For some targets, same pieces were re-used.
